# *Plasmodium falciparum* Drug Resistance Phenotype as Assessed by Patient Antimalarial Drug Levels and Its Association With *pfmdr1* Polymorphisms

**DOI:** 10.1093/infdis/jis747

**Published:** 2012-12-05

**Authors:** Maja Malmberg, Pedro E. Ferreira, Joel Tarning, Johan Ursing, Billy Ngasala, Anders Björkman, Andreas Mårtensson, José P. Gil

**Affiliations:** 1 Malaria Research, Infectious Disease Unit, Department of Medicine Solna; 2 Division of Global Health, Department of Public Health Sciences; 3 Drug Resistance Unit, Division of Pharmacogenetics, Department of Physiology and Pharmacology, Karolinska Institutet, Stockholm, Sweden; 4 Institute of Biotechnology and Bioengineering, Centre of Molecular and Structural Biomedicine, University of Algarve, Faro, Portugal; 5 Mahidol-Oxford Tropical Medicine Research Unit, Mahidol University, Bangkok, Thailand; 6 Centre for Tropical Medicine, Nuffield Department of Clinical Medicine, University of Oxford, United Kingdom; 7 Department of Parasitology, Muhimbili University of Health and Allied Sciences, Dar-es-Salaam, Tanzania; 8 Department of Biological Sciences, The Harpur College of Arts and Sciences, Binghamton University, New York

**Keywords:** *Plasmodium falciparum*, malaria, *pfmdr1*, lumefantrine, artemether-lumefantrine, antimalarials, pharmacokinetics, drug resistance, in vivo

## Abstract

***Background.*** Multidrug-resistant *Plasmodium falciparum* is a major threat to global malaria control. Parasites develop resistance by gradually acquiring genetic polymorphisms that decrease drug susceptibility. The aim of this study was to investigate the extent to which parasites with different genetic characteristics are able to withstand individual drug blood concentrations.

***Methods.*** We analyzed 2 clinical trials that assessed the efficacy and effectiveness of artemether-lumefantrine. As a proof of concept, we used measured day 7 lumefantrine concentrations to estimate the concentrations at which reinfections multiplied. *P. falciparum* multidrug resistance gene 1 (*pfmdr1*) genotypes of these parasites were then correlated to drug susceptibility.

***Results.*** Reinfecting parasites with the *pfmdr1* N86/184F/D1246 haplotype were able to withstand lumefantrine blood concentrations 15-fold higher than those with the 86Y/Y184/1246Y haplotype.

***Conclusions.*** By estimating drug concentrations, we were able to quantify the contribution of *pfmdr1* single-nucleotide polymorphisms to reduced lumefantrine susceptibility. The method can be applied to all long–half-life antimalarial drugs, enables early detection of *P. falciparum* with reduced drug susceptibility in vivo, and represents a novel way for unveiling molecular markers of antimalarial drug resistance.

The evolution and spread of artemisinin-based combination therapy (ACT)–resistant *Plasmodium falciparum* may have drastic consequences on global malaria control and elimination efforts. Development of ACT resistance is likely to start with a decreased efficacy of the long–half-life partner drug, gradually transforming ACT into an unprotected artemisinin-derivative monotherapy. This notion underlies the World Health Organization policy that artemisinin-derivative agents should be exclusively used in combination with other drugs for the treatment of uncomplicated malaria and never together with a failing drug.

In high-transmission areas, the development of resistance against long–half-life partner drugs is likely to occur through the posttreatment selection of less sensitive parasites, as reinfections are exposed to subtherapeutic blood levels of these slowly eliminated drugs. The posttreatment selection of drug resistance–associated single-nucleotide polymorphisms (SNPs) observed after both artemether-lumefantrine [1–3] and artesunate-amodiaquine [4, 5] treatment, involving polymorphisms of the *P. falciparum* multidrug resistance gene 1 (*pfmdr1*; National Center for Biotechnology Information Reference Sequence gene ID 813045), are examples of this.

To identify useful surveillance tools such as genetic markers of resistance against the long-acting partners in ACT, a clear definition of the *P. falciparum* resistance phenotype is needed. Unfortunately, in vivo resistance has been difficult to define with precision. Treatment failure can be due to nonparasitological factors like poor patient drug bioavailability or adherence. Furthermore, the use of biodiversity markers such as *P. falciparum* merozoite surface protein 1 (*pfmsp1*), *pfmsp2*, and glutamate-rich protein (*glurp*) to distinguish between recrudescence (treatment failure) and reinfections may be less reliable than previously expected [6, 7]. As for field in vitro methods, these are only applicable to subsets of infections with appropriate parasitemia and tend to select for high-fitness parasites. We propose a complementary concept for defining molecular markers of in vivo *P. falciparum* susceptibility.

In recent years, we have witnessed important technological developments in the determination of drug levels in blood, using samples collected on filter papers. The increased robustness of these techniques has allowed routine analysis of drug levels of the ACT partner drugs on the seventh day (D7) after treatment initiation as a surrogate marker for drug exposure (ie, area under the concentration-time curve [AUC]) [8]. Long-acting antimalarial drugs (eg, lumefantrine [LUM]) are in their terminal elimination phase at D7 and have a log-linear decrease of drug concentrations [8]. This enables later drug levels to be inferred using the measured D7 concentration and the terminal elimination half-life. The accuracy of the inferred drug concentrations depends on knowledge of the pharmacokinetic characteristics of the target group, especially the terminal elimination rate constant. Estimated drug concentrations can then be correlated to genotypes (SNPs, copy number variation) of recurrent parasites.

As a proof of concept, we correlated polymorphisms in *pfmdr1* with estimated LUM drug concentrations in patients treated with artemether-lumefantrine.

## METHODS

### Clinical Trials

The infections analyzed in this report came from 2 artemether-lumefantrine clinical efficacy/effectiveness trials. Full details of these studies have been reported previously and are summarized in brief in Table 1 [9, 10].

**Table 1.** Description of the Study Populations

Variable	Study 1 (n = 359)	Study 2 (n = 244)	Overall (n = 603)
Reinfection, no. of subjects	170	84	254
Recrudescence, no. of subjects	7	10	17
*pfmdr1* N86 day 0, % of subjects (pure N86/total)^a^	43 (155/357)	49 (115/234)	46 (270/591)
*pfmdr1* N86 reinfection, % of subjects (pure N86/total)	61 (101/166)	79 (65/82)	67 (166/248)
Time to reinfection, d, median (95% CI)^b^	35 (34–36)	28 (25–31)	32 (30–34)

Abbreviations: CI, confidence interval; *pfmdr1, Plasmodium falciparum* multidrug resistance gene 1.

^a^ The present single-nucleotide polymorphism discrimination has been previously published [9, 10].

^b^ The difference in time to reinfection is partly explained by the different follow-up durations in study 1 (56 days) and study 2 (42 days)

Study I was a randomized 2-arm artemether-lumefantrine (AL) clinical trial (efficacy vs effectiveness) among 359 febrile patients <5 years of age (age range, 3–59 months) in the Bagamoyo district of Tanzania [9] (ClinicalTrials.gov identifier ISRCTN69189899). All patients had confirmed *P. falciparum* parasitemia (2000–200 000 asexual parasites/μL) on admission and were followed weekly for 56 days. For the 161 patients with recurrent infection, a second AL treatment was given, and the patient was followed weekly for an additional 42 days.

Study II was a single-arm effectiveness study of the standard 6 dose AL regimen involving 244 subjects from rural Kibaha, Ngeta, and Mwanabwito districts, Tanzania (ClinicalTrials.gov identifier NCT00454961) [10]. Inclusion criteria were identical to those described for study I, with the exception of including individuals with a parasite load of <2000 asexual parasites/μL. The patients were followed weekly for 42 days. For both studies, capillary blood samples were taken at enrollment (D0), at the weekly clinical assessment, and in the event of recurrent parasitemia (R0) and preserved on 3-mm filter paper.

Patients with polymerase chain reaction (PCR)–confirmed reinfections at D7 and onward, with LUM filter paper samples collected on D7 and concentrations successfully measured (see below), were included in the present work. Recrudescences were excluded because they, by definition, have survived treatment with both artemether and LUM. Recrudescent infections therefore represent a different population of parasites. Additional details regarding the patient population are specified in Table 1. Before enrollment, written informed consent was provided by parents or guardians. Both studies were approved by the National Institute for Medical Research, Tanzania, and the Regional Ethics Committee, Stockholm, Sweden.

### Quantification of LUM Concentrations at D7

Seven days after treatment initiation (D7), capillary blood samples were applied on filter papers pretreated with 0.75 M tartaric acid and were stored at −20°C. LUM whole-blood concentrations were measured by solid-phase extraction and liquid chromatography, as described elsewhere [11]. A total of 530 capillary samples were quantified and 34 excluded because measurements were below the limit of detection (100 nM). The within-study assay performance showed a precision (coefficient of variation [CV]) of 6.07%–11.5%.

### Estimation of LUM Concentrations After D7

LUM elimination is in the log-linear phase after D7, and individual drug concentrations can be extrapolated to the point of interest according to the individual pharmacokinetic characteristics. Population estimates were derived from a detailed pharmacokinetic study previously performed in the same setting [12]. The population mean of the terminal elimination half-life used in this work was 80 hours. This was in close agreement with previously published data [13].

The expected drug concentrations on the day of hepatocyte burst were calculated for all patients with reinfections (Equation 1). The estimated day of hepatocyte burst was assumed to occur 7 days before microscopy-based detection of recurrent parasitemia during follow up after AL treatment. This method permitted an in vivo estimate of the reinfecting parasite's ability to multiply under drug pressure.
(1)
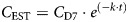

where *C*_EST_ is the estimated LUM blood concentration, *C*_D7_ is the individually measured D7 LUM blood concentration (in nanomoles), *k* is the terminal elimination rate constant set to 0.00865 hours^−1^ [6], and *t* is the time in hours from D7 to estimated hepatocyte burst.

### Data Transformation

Price et al previously defined a venous plasma LUM cutoff concentration of 331 nM (175 ng/mL) to predict recrudescence (treatment failure) with 75% sensitivity and 84% specificity [14]. We converted this value to capillary blood samples, taking into account the hematocrit for our study population, and got an equivalent LUM cutoff concentration of 328 nM (Supplementary Material). Thus, LUM concentrations >328 nM at D7 are defined here as the threshold of exposure of the parasites to an adequate AUC.

### Molecular Analysis

The *pfmdr1* N86Y status of every recurrent parasitemia (R_0_, initial day of microscopy-determined recurrent parasitemia), previously determined through PCR–restriction fragment length polymorphism analysis [9, 10], was reanalyzed and confirmed through reamplification and direct PCR amplicon sequencing. The *pfmdr1* Y184F and D1246Y SNPs were analyzed by PCR amplicon sequencing. One patient, who experienced a second recurrent infection, with estimated LUM blood concentrations of >550 nM, was also added to the analysis. The PCR success rates for analysis of codon 86, 184, and 1246 were 97% (259 of 267), 92% (245 of 267), and 90% (241 of 267), respectively. The presence of *pfmdr1* copy number variation, previously associated with in vivo artemether-lumefantrine response [14, 15], has been previously tested [9], with only 1 infection identified as carrying 2 copies (86Y). Reinfections and recrudescences were distinguished using previously published stepwise genotyping of *pfmsp2*, followed by *pfmsp1* and *glurp* [9, 10]. For primer sequences, see Supplementary Table 1.

### Statistical Analysis

The statistical analyses were done using Stata v.12 and SigmaPlot 11. Statistical significance was defined as a *P* value of < .05. The 95% confidence intervals were computed in STATA v.12, for binomial variables, and by SigmaPlot 11, for survival data. Normally distributed continuous data were analyzed with the Student *t* test. A Mann–Whitney rank sum test was used to compare estimated LUM blood concentrations for different genotypes. Mixed infections with the presence of both alleles were excluded from analysis. Only pure infections were analyzed for *pfmdr1* haplotypes, and rare haplotypes present in <3 infections were excluded.

## RESULTS

**Figure 1.**
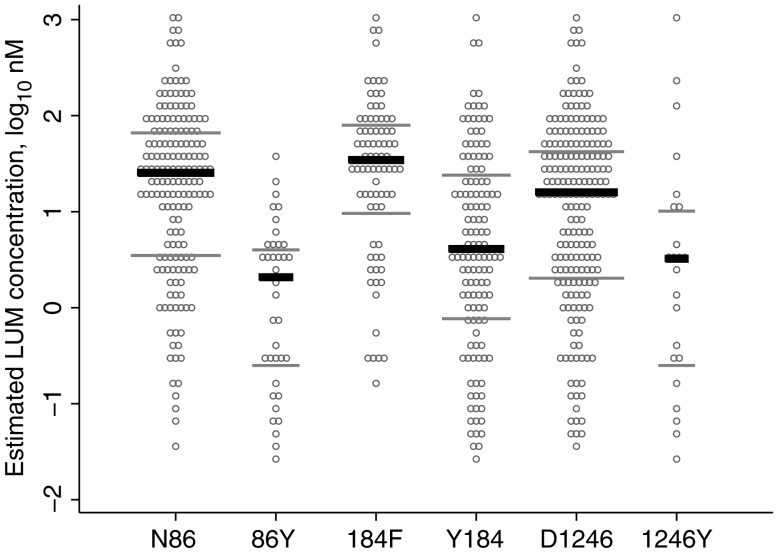
Estimated lumefantrine (LUM) concentrations for reinfecting *Plasmodium falciparum* carrying different *pfmdr1* single-nucleotide polymorphisms (SNPs) at codons 86, 184, and 1246. Each reinfection is represented 3 times, once for each SNP. Only pure infections (concerning the *pfmdr1* SNPs) were included in the analysis. According to the Mann–Whitney rank sum test, there was a significant difference between N86 and 86Y (*P* < .001), 184F and Y184 (*P* < .001), and D1246 and 1246Y (*P* = .006; Table 2). Black lines, median values; grey lines, interquartile ranges.

Reinfecting parasites carrying *pfmdr1* N86, 184F, or D1246 pure alleles were able to survive at significantly higher median estimated LUM blood concentrations, compared with parasites harboring their alternative alleles (Figure 1 and Table 2). The largest difference was observed for the N86Y SNP, with concentrations of 25.4 nM versus 2.08 nM (a 12.2-fold difference) between the N- and the Y-carrying parasites. For *pfmdr1* Y184F and D1246Y, the corresponding differences were 4.09 nM versus 34.5 nM (an 8.4-fold difference) and 15.9 nM versus 3.23 nM (a 4.9-fold difference), respectively.

**Table 2.** Estimated Median Lumefantrine (LUM) Blood Concentrations for Different *Plasmodium falciparum* Multidrug Resistance Gene 1 (*pfmdr1*) Single-Nucleotide Polymorphisms (SNPs) and Haplotypes

*pfmdr1*	No.	LUM *C*_EST_, nM	Interquartile Range	*P*
SNP				
N86	166	25.4	3.85–72.7	< .001
86Y	37	2.08	0.248–4.43	
184F	80	34.5	10.5–87.5	< .001
Y184	127	4.09	0.879–25.4	
D1246	195	15.9	2.26–46.4	.006
1246Y	23	3.23	0.293–10.9	
Haplotype				
NFD	64	31.4	10.5–76.1	
NYD	63	15.8	2.53–46	.045
YYY	15	2.16	0.293–3.77	≤.001
YYD	15	0.678	0.108–3.87	≤.001

Abbreviation: C_EST_, estimated LUM concentration.

Combinations of pfmdr1 polymorphisms at codon N86Y, Y184F, D1246Y. Mann–Whitney rank sum test was used to compare estimated lumefantrine concentrations between SNPs and the NFD haplotype against other haplotypes.

**Figure 2.**
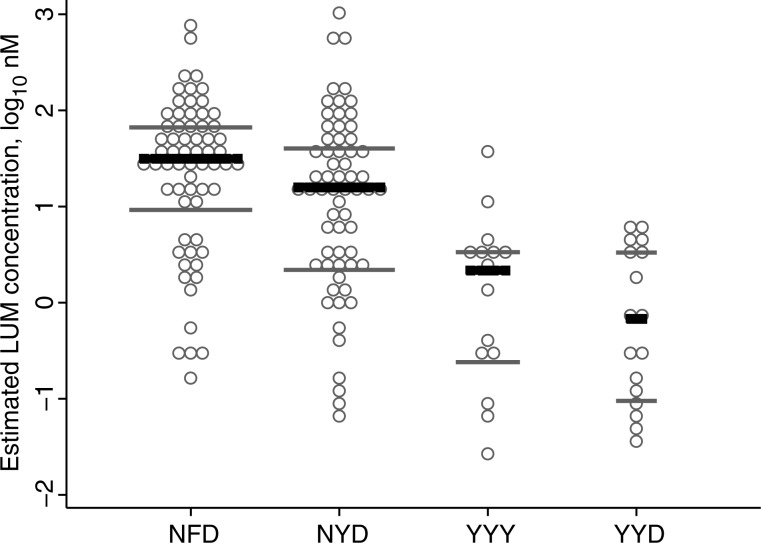
Estimated lumefantrine concentrations for reinfecting *Plasmodium falciparum* carrying different *pfmdr1* haplotypes at codons 86, 184, and 1246. Each open circle represents a reinfection. Only haplotypes with ≥3 observations were considered for analysis. Median values were 31.4 nM (interquartile range [IQR], 10.5–76.1 nM) for NFD, 15.8 nM (IQR, 2.53–46.0 nM) for NYD, 2.16 nM (IQR, 0.293–3.77 nM) for YYY, and 0.678 nM (IQR, 0.108–3.87 nM) for YYD (Table 2). Black lines, median values; grey lines, interquartile ranges.

*P. falciparum* with the N86/184F/D1246 haplotype was statistically significantly less sensitive than *P. falciparum* with the alternative haplotypes 86Y/Y184/1246Y (31.4 nM vs 2.16 nM [a 14.5-fold difference]; *P* < .001) and 86Y/Y184/D1246 (31.4 nM vs 0.678 nM [a 46.3-fold difference]; *P* < .001; Figure 2 and Table 2). There was no significant correlation between *pfmdr1* haplotypes and D0 parasitemia (as a proxy marker of fitness)

The highest estimated LUM concentration that reinfecting parasites carrying N86 versus those carrying 86Y could withstand differed by a factor of 35 (1184.3 nM and 34.3 nM, respectively; Figure 1). The influence of Y184F and D1246Y SNPs on drug susceptibility is less clear-cut, with the “sensitive” Y184 and 1246Y parasites able to withstand the highest drug levels (1184.3 nM and 1081.5 nM, respectively.

There was a distinct subset of 8 parasites that were able to grow at estimated LUM concentrations of >550 nM, whereas no other parasites grew at concentrations >300 nM (Table 3). The subset represented 4.57% (8 of 175; 95% confidence interval, 1.99%–8.81%) of the reinfections occurring up to 35 days after treatment initiation. These least susceptible parasites all carried the *pfmdr1* N86 allele and had LUM D7 levels 1.8–10.3-fold higher than 328 nM, confirming adequate treatment and bioavailability.

**Table 3.** Reinfecting *Plasmodium falciparum* Able to Grow at Estimated Lumefantrine (LUM) Concentrations of >550 nM

Parasite Code	Study	LUM *C*_EST_, nM	LUM *C*_D7_, nM	*pfmdr1* N86Y	*pfmdr1* Y184F	*pfmdr1* D1246Y
F147	I	1184	1184	N	Y	D
11129	II	1081	1081	N	F	Y
F26	I	706	1070	N	F	D
F63	I	678	678	…	…	D
11066	II	581	581	N	Y	D
9096	II	794	3397	N	F	…
F202	I	565	2416	N	Y	D
F13	I	558	2388	N	F	D

Polymorphisms in *pfmdr1* at codon N86Y, Y184F, and D1246Y were analyzed on the day of recurrent parasitaemia.

Abbreviations: C_D7_, measured LUM concentration 7 days after treatment initiation; C_EST_, estimated LUM concentrations; D, aspartic acid; F, phenylalanine; N, asparagine; Y, tyrosine; –, unsuccessful polymerase chain reaction analysis.

There were no significant differences in D7 concentration between patients who were reinfected with one of these “least susceptible parasites” and those who were adequately treated and then experienced recrudescence (*P* = .113; Supplementary Table 2).

## DISCUSSION

The present study allowed an estimate of in vivo susceptibility to LUM by *P. falciparum* and its association with *pfmdr1* alleles. The data provide evidence that the observed post-AL treatment selection of *pfmdr1* alleles is associated with a significant decrease in LUM susceptibility. This reinforces the hypothesis that *pfmdr1* is a central player in *P. falciparum* resistance to LUM.

We found that *pfmdr1* N86, 184F, and 1246D were associated with reduced LUM susceptibility, compared with the alternative 86Y, Y184, and Y1246 alleles. When analyzed as haplotypes, the NFD haplotype were able to withstand estimated LUM concentration 15-fold higher than those with the YYY haplotype. This is in line with previous in vivo and in vitro work [1, 2, 16]. Clinical isolates from Kenya with *pfmdr1* N86 had a 2.9-fold higher median LUM median inhibitory concentration than the 86Y allele in vitro [16]. In line with the Kenyan results, we found a 12.2-fold difference between these 2 SNPs in vivo. Haplotype analysis showed a trend of decreased LUM susceptibility, in the order of NFD, NYD, YYY, and YYD. This suggests a gradually acquired tolerance, starting with N86, followed by the combination of N86 + D1246 and, thereafter, the combination of N86 + 184F + D1246. This might be comparable with the selection of SNPs in *P. falciparum* dihydrofolate reductase (*pfdhfr*) associated with a stepwise decrease in susceptibility to sulfadoxine-pyrimethamine [17]. The D0 parasitemias were similar irrespective of haplotype, supporting the hypothesis that differences in drug tolerability is a measure of reduced drug susceptibility as opposed to fitness.

An important question is whether we have identified LUM-resistant parasites. We identified *P. falciparum* parasites able to survive at levels near or above blood drug concentrations of 1 µM. Such capacity to withstand drug pressure means that these parasites are able to start proliferating just 2 days after completion of the AL treatment, when the LUM blood levels drop to these levels [18]. Irrespective of exact concentrations, taking into account sampling errors, such a collapse of protection capacity is probably paving the way for the emergence of fully resistant parasites (if they are not already present). This is in agreement with a previously proposed model describing AL-driven *pfmdr1* SNP selection in Africa [18]: the parasite is developing its way of “climbing” the pharmacokinetic curve. As discussed above, the N86 allele seems to be fundamental to—albeit not sufficient for—this process, to which other *pfmdr1* SNPs (184F and D1246) and additional, as-yet-unveiled genetic changes add.

The proposed in vivo genotype/phenotype association method has the advantage of being independent of constraints associated with the definitions of recrudescence and clinical failure [6, 19], drug bioavailability, and other issues not directly related to parasitological drug sensitivity. The method provides an in vivo estimate of the capacity of parasites to evade drug action. It has an in-built high specificity, since no sensitive parasites are expected to be detected at high blood drug levels. This high specificity is expected to be an important factor for the unambiguous identification of tolerant/resistant *P. falciparum*. The method will underestimate the proportion of parasites with reduced drug susceptibility, because such parasites, as well as fully susceptible parasites, will thrive when drug concentrations are low. The method will thereby be prone to false-negative findings. Further meta-analysis of clinical trials using this method will increase the possibility to reliable identify resistant parasites and resistance-associated SNPs.

In this work, we have benefited from a previous detailed pharmacokinetic study performed in the same area on a similar study population [12]. This provided the necessary data for inferring LUM concentrations beyond D7. Such pharmacokinetic data from the specific target populations is generally not available in malaria settings. We suggest that for the application of the proposed concept, future ACT efficacy/effectiveness trials should preferably include at least 2 drug level assessment points (eg, at D7 and D14), thereby defining an individual slope of drug elimination for each patient. This would overcome the limitation in this study of using the mean population terminal elimination half-life rather than individual values to extrapolate individual D7 levels to the time of hepatocyte burst. Because of the pharmacokinetics of LUM, we expect a significant number of patients to show quantifiable concentrations of this antimalarial 2 weeks after treatment initiation [20]. Additionally, recent developments in liquid chromatography–mass spectrometry [21] promise improvements of least 1 order of magnitude (ie, down to 1–10 nM) in lower limits of detection. This will make the determination of the D14 LUM concentrations feasible in a large majority of patients.

Our proof of concept was applied to artemether-lumefantrine clinical trials. This method is, however, equally valuable for studying emerging resistance toward all ACT long-acting partner drugs. The use of drug concentrations can help in accurate interpretation of clinical trial outcomes and will also give an improved definition of the phenotype associated with reduced susceptibility. The concept can also be used to identify genotypes associated with reduced susceptibility to *Plasmodium vivax*, where ex vivo/in vitro work is limited.

In conclusion, we present a new concept using D7 drug concentrations and pharmacokinetic data to estimate the drug concentrations that parasites withstand in vivo. We found that reinfecting parasites with the *pfmdr1* N86/184F/D1246 haplotype were able to withstand LUM blood concentrations 15-fold higher than parasites with the *pfmdr1* 86Y/Y184/1246Y, supporting the role of *pfmdr1* in LUM susceptibility. Our method to correlate drug concentrations and genotypes is applicable to all antimalarial drugs, can contribute to the early detection of reduced drug susceptibility, and represents a novel way for unveiling molecular markers of antimalarial drug resistance.

## Supplementary Data

Supplementary materials are available at *The Journal of Infectious Diseases* online (http://jid.oxfordjournals.org/). Supplementary materials consist of data provided by the author that are published to benefit the reader. The posted materials are not copyedited. The contents of all supplementary data are the sole responsibility of the authors. Questions or messages regarding errors should be addressed to the author.

Supplementary Data
