# Supplementary material for: Plasmodium falciparum Drug Resistance Phenotype as Assessed by Patient Antimalarial Drug Levels and Its Association With pfmdr1 Polymorphisms
Source: J Infect Dis. 2012 Dec 5;207(5):842–7. doi: 10.1093/infdis/jis747 (PMC3563306; doi:10.1093/infdis/jis747)
Supplement: Supplementary Data [file supp_207_5_842__index.html]

Plasmodium falciparum drug resistance phenotype as assessed by patient antimalarial drug levels and its association with pfmdr1 polymorphisms — Plasmodium falciparum Drug Resistance Phenotype as Assessed by Patient Antimalarial Drug Levels and Its Association With pfmdr1 Polymorphisms — Plasmodium falciparum Drug Resistance Phenotype as Assessed by Patient Antimalarial Drug Levels and Its Association With pfmdr1 Polymorphisms — Supplementary Data 

# *Plasmodium falciparum* Drug Resistance Phenotype as Assessed by Patient Antimalarial Drug Levels and Its Association With *pfmdr1* Polymorphisms

## Supplementary Data

Supplementary Data

**Files in this Data Supplement:**

- Supplementary Data - Docx file
