# Supplementary material for: Plasmodium falciparum Drug Resistance Phenotype as Assessed by Patient Antimalarial Drug Levels and Its Association With pfmdr1 Polymorphisms
Source: J Infect Dis. 2012 Dec 5;207(5):842–7. doi: 10.1093/infdis/jis747 (PMC3563306; doi:10.1093/infdis/jis747)
Supplement: Supplementary Data [file supp_jis747_jis747supp.docx]

***Transformation of lumefantrine concentration cut-off associated with protection against recrudescence***

It has been previously reported that a LUM D7 concentration above 175 ng/ml (331nM) (measured in venous blood) was associated with a significant decrease in the probability of a recrudescent malaria infection to occur [1]. In our study we have evaluated LUM levels in capillary plasma, so in order to use this threshold in our studies we convert the cut-off value of 331nM to its corresponding value in capillary plasma based on an established method [2]. A haematocrit value (HCT) of 30% (i.e. 3 times the median haemoglobin-value, measured by HemoCue^®^ at day of re-treatment in Study I) and a total distribution of 8.2% of LUM into red blood cells (LUM_RBC%_) was taken into account for a modified formula converting venous plasma concentrations into capillary blood concentrations: Capillary blood concentration = Capillary plasma concentration _*_ ((1-HCT)/(1-LUM_RBC%_))[3]. The equivalent value of 331nM LUM was estimated to be 328 nM when measured in capillary blood.

1. Price RN, Uhlemann AC, van Vugt M, et al. Molecular and pharmacological determinants of the therapeutic response to artemether-lumefantrine in multidrug-resistant Plasmodium falciparum malaria. Clinical infectious diseases : an official publication of the Infectious Diseases Society of America **2006**; 42:1570-7.

2. van Vugt M, Ezzet F, Phaipun L, Nosten F, White NJ. The relationship between capillary and venous concentrations of the antimalarial drug lumefantrine (benflumetol). Trans R Soc Trop Med Hyg **1998**; 92:564-5.

3. Colussi D, Parisot C, Legay F, Lefevre G. Binding of artemether and lumefantrine to plasma proteins and erythrocytes. Eur J Pharm Sci **1999**; 9:9-16.

**Table S1.** Primer sequences.

|  | |  |  |
| --- | --- | --- | --- |
| **Amplified *pfmdr1* codons** | **1st amplification (5' - 3')** | **2nd amplification (5' - 3')** | **Sequencing primers (5' - 3')** |
| N86Y | Fw-AAGAGGTTGAAAAAGAGTTGAAC | Fw-AGAGTACCGCTGAATTATTTAG |  |
|  | Rev-ATTTCGTACCAATTCCTGAACT | Rev-CCTGAACTCACTTGTTCTAAAT |  |
| 1-292 | Fw - GTGTACATAGCTTATTTCATTTATA | Fw - GCTTATTTCATTTATAAGATTTA |  |
|  | Rev - ATTGATTCGTTGCACTATTTA | Rev - CGAATGCATAAGAAACTAA | Rev - CGAATGCATAAGAAACTAA |
| 974-1283 | Fw - GATGATGAAATGTTTAAAGATCC | Fw - GATGATGAAATGTTTAAAGATCC | Fw - GATGATGAAATGTTTAAAGATCC |
|  | Rev - TTGTCCACCTGATAAGCTTT | Rev - ACACGTTTAACATCTTCCAAT | Rev - ACACGTTTAACATCTTCCAAT |

**Table S2. Details of recrudescent infections.**

| **Code** | **Study** | **LUM C_D7_** **(nM)** | **R0 (day)** | ***pfmdr1* N86Y** | ***pfmdr1* Y184F** | ***pfmdr1* D1246Y** |  |
| --- | --- | --- | --- | --- | --- | --- | --- |
| F47 | I | 2143 | 28 | N | F | D | Above the  328nM cut-off |
| F93 | I | 857 | 32 | N | F | D |  |
| 9076 | II | 791 | 21 | N | Y | D |  |
| 10014 | II | 779 | 28 | N | Y/F | D |  |
| F118 | I | 713 | 28 | N | F | D |  |
| F2 | I | 366 | 38 | Y | Y | Y |  |
| 11043 | II | 330 | 35 | N/Y | Y | D/Y |  |
| F46 | I | 291 | 28 | N | F | D | Below the  328nM cut-off |
| 11146 | II | 287 | 14 | N | F | D |  |
| Y86 | I | 284 | 21 | N | Y | D |  |
| 11068 | II | 257 | 14 | N | F | D |  |
| F68 | I | 164 | 35 | N | Y | D |  |
| 9059 | II | 108 | 14 | N | F | D |  |
| 9017 | II | 0 | 7 | N | - | D |  |
| 9030 | II | 0 | 21 | N | Y/F | D |  |
| 9048 | II | 0 | 7 | N | Y | D |  |
| 9080 | II | 0 | 21 | N | Y | D |  |

LUM C_D7_ is measured LUM blood concentrations 7 days after treatment initiation, R0 is day of recurrent parasitaemia, *P. falciparum multidrug resistance gene 1* (*pfmdr1*) N86Y, Y184F and D1246Y are polymorphisms at codon 86, 184 and 1246 analysed at R0 day (N= Asparagine, Y= Tyrosine, F= Phenylalanine, D=Aspartic acid, – denotes unsuccessful PCR).
